# Normative and Equated Data of the Original and Basic Versions of the Montreal Cognitive Assessment among Community Dwelling Saudi Arabians

**DOI:** 10.1155/2021/5395627

**Published:** 2021-01-06

**Authors:** Taim A. Muayqil, Nada K. Alamri, Awyshah M. Alqahtani, Sarah S. Julaidan, Raya Alsuhaibani, Ibrahim Nafisah, Walid A. Alkeridy, Bandar N. Aljafen, Mohammed H. Alanazy

**Affiliations:** ^1^Neurology Unit, Department of Medicine, College of Medicine, King Saud University, Riyadh, Saudi Arabia; ^2^Department of Statistics and Operations Research, College of Science, King Saud University, Riyadh, Saudi Arabia; ^3^Department of Medicine, College of Medicine, King Saud University, Riyadh, Saudi Arabia; ^4^Department of Medicine, Geriatric Division, University of British Columbia, Vancouver, Canada

## Abstract

**Introduction:**

Currently, there are standard and basic versions of the MoCA, the latter designed for those with lower educational achievements. Community-based normative data on these versions of the MoCA from Arabic populations are deficient, and there is little data demonstrating how both scales perform in comparison. We aim to obtain normative performances from both versions and equate the measures of both scales.

**Methods:**

Community-based recruitment of healthy volunteers ≥ 18 years of age. Participants underwent testing with both versions. Demographic data was collected with regard to age, gender, years of education, diabetes, and hypertension. Regression analysis was performed to determine significance of variables, and the circle-arc equating method was used to equate the two scores from each scale.

**Results:**

311 participants were included in the study. The mean (sd) age was 45.8 (15.96), females were 184 (59.16%), and the duration of education was 12.7 (5.67) years. The mean scores on the MoCA-A and MoCA-B were 21.47 (4.53) and 24.37 (4.71) (*P* < 0.0001), respectively. Multivariate regression showed significance of age and years of education in both versions (both variables with *P* < 0.0001). Correlation coefficient between the two scales was 0.77 (*P* < 0.0001). The largest equated difference between both MoCA versions was four points in those scoring from 10-20 on the MoCA-A.

**Conclusion:**

We present normative data from a large Saudi Arabian community-based sample with two different MoCA tests, and an equating graph is presented to determine the corresponding expected performance between the two scales.

## 1. Introduction

Cognitive impairment can occur at any age due to a plethora of diseases and disturbances. The assessment of cognitive impairment typically starts at the level of the clinician who then applies screening tools to help determine its presence or identify its subtype. The Montreal Objective Cognitive Assessment (MoCA) for the past fifteen years has aided significantly in the diagnosis and identification of cognitive disorders, particularly in geriatric patients [1]. Its popularity also stems from it being a brief screening tool that can quickly give an impression about a patient's cognitive state.

Since the MoCA became a well-established measurement tool for identifying dementia and mild cognitive impairment (MCI) [1], it has become available in multiple versions for repeat testing and in multiple languages. Obtaining normative neuropsychological data that is unique to a particular culture is crucial [2, 3] in order to avoid either over or under identification of cases. Many countries have determined the normative values and even the diagnostic accuracy for diagnosing MCI or dementia [4–10]. While correcting for education and age can help in interpreting the results [3, 11], there are still likely to be some unique cultural factors that might go unadjusted, urging the need to develop normative data for each country and even sometimes for different ethnicities within a single region [3, 12, 13]. Multiple studies, primarily those that were community-based or equated the MoCA with the Mini Mental State Exam (MMSE), have proposed reconsiderations for the diagnostic cut-offs [3, 14–18].

While most data is available on the original MoCA version, some emerging studies have demonstrated that a more recently developed basic version of the MoCA to be a similarly valid tool [19, 20] in those with lower levels of education. The MoCA is not only more reliable in identifying cognitive impairment than the MMSE [21] in elderly, but also has applicability outside of the geriatric population. It is becoming popular in determining cognitive changes in all adult age groups for various systemic and neurological conditions. Prominent examples include substance abuse [22], systemic lupus erythematosus [23], epilepsy [24], early Parkinson's disease [25], cardiac [26], and cerebrovascular diseases [27]. This recent expansion of use warrants obtaining normative data from all adult age groups. More specific to Saudi Arabia, determination of norms is necessary given an approximate 7% illiteracy rate in general, with much higher proportions in the geriatric population [28]. Furthermore, with the expected worldwide increase in dementia [29], there is a pressing need to study these scales in different societies. Our objective was to demonstrate real-world normative community data based on the performance on both the standard and basic versions of the Arabic MoCA in a healthy Saudi Arabian sample and equate both measures in a population comprising of different individuals with varying ages and different education levels.

## 2. Methods

### 2.1. Participants

This was a cross-sectional study conducted at King Saud University, Riyadh, Saudi Arabia. Participants were randomly recruited from the community by convenience sampling or snowball methods and also from patient companions or patients who met the inclusion and exclusion criteria during their visit to the university hospital for various services. Recruitment was from December 2017 to September 2018. Healthy participants, including those with controlled diabetes or hypertension and no history of complications, above 18 years of age were included. We excluded those who had chronic complicated systemic diseases, neurological diseases, suspicion of cognitive problems, or focal neurological deficits determined from participants' medical history. Also excluded were those with any psychiatric condition requiring treatment, any surgical intervention seven days prior to the date of the interview, or the use of any medication that could influence cognition. Assuming a normally distributed population, our goal was to obtain at least 50 participants in each age decade in order to obtain representable normative data [30]. This study was approved by the Institutional Review Board at the College of Medicine, King Saud University, Riyadh, Saudi Arabia.

### 2.2. Procedure

Demographic data on age, gender, and years of education were collected in addition to the presence or absence of controlled diabetes and hypertension. Cognitive assessment was done using the standard and basic Arabic versions of the Montreal Objective Cognitive Assessment (MoCA) test, referred to as MoCA-A and MoCA-B, respectively, both tests were downloaded from the website http://www.mocatest.org, and there is currently only one version of each in Arabic. Each version is a paper test established to assess seven subdomains of cognition with the possible maximum score of 30. All participants were interviewed by a medical student who was trained by a specialist in the neurocognitive field. Participants were pseudorandomized so that half started with version A and the other half started with version B, for those who completed both versions. Each version took 10–15 minutes to be completed. Scoring and administration of the scale were according to instructions on the website. A pilot study was done on 15 participants, and minor modifications were made to suit the culture and dialect with permission from http://mocatest.org for our research purposes. One of the recall items on the MoCA-A was “the clove plant قرنفل” changed into “Basil ريحان” a more familiar plant. To be in line with the local dialect, in the language section, repetition was correct if the subject used an alternate function word “على” instead of “عن” in the second sentence (ابو نسيب زار جاره واطمأن عن صحته). In the MoCA-B version, the currency in the calculation test was changed from Egyptian Pounds to the local Saudi Riyals. In the Abstraction section, the term used for “Boat مركب” was not very familiar in the regional dialect and also served as a cue to the answer; therefore, the term was replaced with “Ship سفينة.”

### 2.3. Analysis

**Figure 1:**
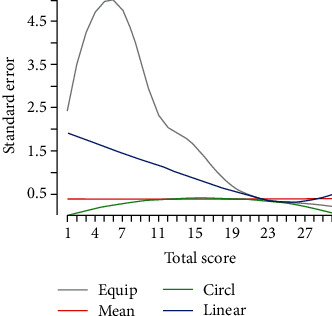
Standard errors graph of equating methods.

Exploratory statistical analysis was performed on basic demographic variables to estimate means and proportions where appropriate. Age and years of education were categorized to estimate performance on each MoCA version within each of these categories. Proportion of participants who completed each subtest correctly was also estimated for both versions. Univariate analyses performed on each version of the MoCA where the total raw score was the dependent variable and age, years of education, gender, diabetes, or hypertension were the independent variables. Significant variables were then included in the multivariate analysis, and Stata 15 software was used. Equating procedure of scores was done using the equate package in *R* [31] after log transformation. Four equating measures: mean, linear, equipercentile, and circle arc were applied, with the circle arc showing the lowest standard errors compared to the other methods after bootstrapping (Figure 1) and was therefore the equating measure of choice.

## 3. Results

**Figure 2:**
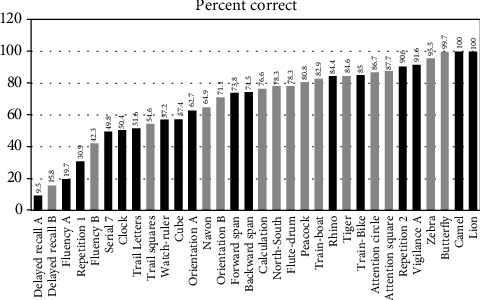
Percent correct obtained in each subtest of both MoCA versions.

The mean (sd) age was 45.8 (15.96) ranging from 18 to 80 years of age, and 184 (59.16%) were female. The mean duration of education was 12.7 (5.67) years. The mean score on the MoCA-A obtained from 275 participants was 21.47 (4.53), and the mean on the MoCA-B obtained from 286 participants was higher at 24.37 (4.71) (*P* < 0.0001). Details on demographic variables by gender are presented in Table 1. Performance on each version of the test according to different ages and durations of education categories are shown in Tables 2. On the MoCA-A, 220 (80%) scored below the proposed cut-off of <26 for cognitive impairment [1] and on the MoCA-B 167 (58.4%) scored below a proposed cut-off of ≤26 [19]. The Pearson correlation coefficient between the two scales was 0.77 (*P* < 0.0001). The percentage of individuals who obtained a complete score within each subtest is demonstrated in Figure 2.

**Figure 3:**
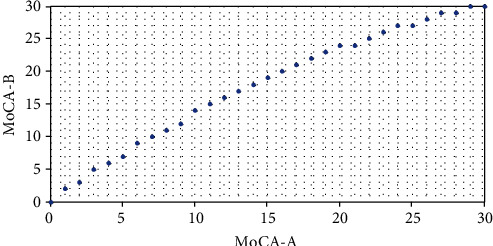
Equated scores between both MoCA versions.

Univariate regression for each MoCA version was carried out involving the variables age, years of education, gender, diabetes, and hypertension. For the MoCA-A, the significant variables were age (*P* < 0.0001), years of education (*P* < 0.0001), diabetes (*P* < 0.0001), and hypertension (*P* < 0.0001). Gender was nonsignificant (*P* = 0.8). For the MoCA-B, age, years of education, and hypertension were significant, each (*P* < 0.0001). Diabetes was significant (*P* = 0.001) and gender nonsignificant (*P* = 0.08). Multivariate regression, including only the statistically significant variables from the univariate analysis, showed that only age and years of education were significant variables in both the MoCA-A (*F* = 54.38, *P* < 0.0001, adjusted *R*^2^ = 0.44) and the MoCA-B (*F* = 64.1, *P* < 0.0001, adjusted *R*^2^ = 0.47) (Table 3). Every one year increase in age was associated with a point decrease of 0.06 and 0.07 on the MoCA-A and MoCA-B, respectively. Conversely, every one year increase in education was associated with 0.46 and 0.43 points increase on the MoCA-A and MoCA-B, respectively.  Figure 3 demonstrates the equating of both test scores based on 250 participants who completed both assessments.

## 4. Discussion

In this study, we have demonstrated the results of two versions of the MoCA which differ in difficulty in a Saudi Arabian sample with an equating measure of the two scales and presented normative community-based data spanning different ages and levels of education. Among variables influencing performance, age and education appear to be the most important. This is consistent with the performance on most cognitive assessment scales [32]. Performance improves with higher levels of education and decreases with increasing age. This pattern has been repetitively reproduced in previous MoCA studies in different countries and ethnicities [4–10]. While not finding differences according to gender is also common [4, 9, 33, 34].

The inclusion of a wide spectrum of ages and education levels was helpful in this study in order to help better understand the spectrum of performance. This helped clarify that the lower scores seen here, in comparison to other language versions, are likely related to factors other than just age and education. We suspect that these factors are primarily from effects one's culture might have while undergoing a cognitive assessment. Previous literature has demonstrated an effect of culture on the MoCA and other neuropsychological assessments in ethnic minority groups, such as African Americans and Hispanics in the United States [3, 13, 18] and North African [12] and Turkish [35] immigrants in Europe. African Americans have been found to have lower scores in various cognitive tasks, such as those involving naming, pattern matching, and money management, and these findings were regardless of age and education [36]. Several explanations for this effect have been provided, namely, the level of acculturation [37]. Higher degrees of acculturation indicate a larger degree of incorporation into current mainstream culture, and those who were less integrated in the form of language and other social behaviors performed lower on many scales of cognitive tasks [13]. This explains why using the MoCA, a tool designed and implemented in a different society and culture, has yielded lower results in the current sample. Additionally, the experienced rapid societal development in Saudi Arabia can result in a cultural gap between adjacent generations, which might further contribute to this phenomena. Many studies have established differences among ethnicities, even if these ethnic groups live within the same country or region [36]. The quality of education is similarly an important factor in cognitive performance and not just the duration of formal education [3, 38]. In minority groups, one has to be conservative when associating performance with years of education as the actual level of performance might be lower than expected [38]. Western neuropsychological studies, that included measures of participants' quality of education, have shown differences in neuropsychological test performance likely explained by a disparity in education quality between ethnic minorities and those from the mainstream culture after correcting for years of education and age [13, 38]. Language ability has been related to acculturation and in cognitive performance differences between ethnic groups [39, 40]. Reading ability is a major variable to consider since it is a marker of the quality of education, and it has been found to associate with many of the discrepancies on neuropsychological assessments [13]. Reading ability inconsistent with the individuals level of education had a prominent association with neuropsychological performance in one study and was more pronounced than ethnicity, highlighting the role education quality that has on these assessments [41]. Other factors to consider are anxiety or a sense of threat during test taking [42, 43], test-wiseness [38], or stereotype threat [44]. The latter being a situation where performance may be affected due to a concern by the test taker that the assessment will confirm a negative stereotype [45], particularly experienced by elderly patients, individuals from rural regions, or those with lower years of education. Stereotype threat occurs in young and old and has been demonstrated to negatively impact performance on neuropsychological assessments [46]. It has been demonstrated experimentally by functional neuroimaging that emotional regulation areas, namely, the anterior cingulate cortex, were activated when stereotypes were suggested to test participants, instead of the activation of the regions required to carry out the specified cognitive task [47]. Despite these effects of culture on different ethnicities, tests that measure an individual's learning ability do not show much difference [48] between them. Additionally, the rate of cognitive decline in ageing populations appears to be similar among different races [49].

As expected, there was a high correlation between the two scales, and the scores of the MoCA-A were significantly lower, consistent with the nature of MoCA-B being a relatively less demanding cognitive assessment. Equating the two scales is useful for interpreting follow-up results when each assessment was done with a different scale and for clinicians to become accustomed to the relatively newer assessment. The largest equated difference between both MoCA versions was four points in those scoring within 10-20 points on the MoCA-A, which was not as large as in studies that equated the original MoCA with the MMSE [15, 21, 50, 51]. Additionally, the ceiling effect was much smaller than that seen when equating with the MMSE [15, 50, 51]. The MMSE relies heavily on orientation and is less capable of detecting MCI in general [52]. These findings in the equating measure propose that the MoCA-B, despite being an easier task, is likely capable of detecting impairments in various cognitive domains with better sensitivity than the MMSE. However, data with actual patients suffering from cognitive impairment will be required to verify this. The majority of participants in both MoCA versions scored below the established cognitive impairment cut-offs [1, 19], and this would remain true even if a one point correction was applied, requiring the consideration of a different cut-off for cognitive impairment in this community.

In comparison to studies that included minorities and differing ethnicities [3, 53, 54], the overall pattern of item difficulty was similar, with recall of all 5 words being amongst the most difficult. This is an important finding to consider when interpreting the delayed recall responses, reviewing the performance of cued recall gives additional unscored information on the likely cause of the memory impairment. The abstraction item about measurement tools, cube copy, repetition of the long sentence, trail making test, and clock drawing was difficult in general. The cube copy has been previously found to be one of the more difficult items, as well as the “measurement tools” from the abstract thought assessment question [55]. Performances on fluency were much lower than other studies, and this might be related to letter properties and the application of the rules of the test. In Arabic, many adjectives that start with the letter “ف” (phonemic equivalent of “F”) could also serve as a person's name, thereby limiting the amount of words that can be produced with this letter while respecting the “no names” rule. Fruit fluency on MoCA-B was performed much better; however, this cannot serve as an alternative due to the different cerebral localizations for these tests.

While we report on a reasonable number of participants with variation in age and years of education, there are limitations to consider; the volunteer recruitment method might have attracted participants, who had concerns about their cognition, despite falling within the study inclusion and exclusion criteria. It is also important to point out that this is a community sample that did not have detailed neuropsychological screening to rule out occult cognitive problems, as was done in the original MoCA study [1]. This is likely similar to the results found in those with lower cut-offs in other community-based studies [6, 9, 34].

In summary, we presented normative data from community members living in Riyadh for two versions of the MoCA and equated both scales. While both versions of the MoCA studied here are useful tools for detecting cognitive impairment, future research in patients with impairments will be required to determine appropriate diagnostic cut-offs. The regular cut-offs in the Arabic version cannot apply given that there are likely various factors, apart from age and education, that might limit the application of a directly translated version. Future studies should be directed to broadening the scope of cognitive tasks that can be used effectively in the Saudi society along with the inclusion of patients with differing levels of cognitive impairment.

## Figures and Tables

**Table 1:** Mean (SD) are provided for age, years of education, and MoCA scores. The remaining variables are numbers (percentages).

	Male		Female	
	*N*	Mean (SD) or *n* (%)	*N*	Mean (SD) or *n* (%)
Age	127	48.95 (17.35)	184	43.62 (14.58)
Years of education	125	13.76 (5)	180	12.03 (6.01)
Right handed	126	122 (96.8%)	181	172 (95%)
Diabetes	127	31 (24.4%)	184	36 (19.6%)
Hypertension	126	31 (24.6%)	184	33 (17.9%)
MoCA-A score	121	21.39 (4.28)	154	21.53 (4.73)
MoCA-B score	120	23.81 (4.57)	166	24.78 (4.79)

**Table 2:** Scores on the MoCA-A and MoCA-B versions according to age and education category.

		MoCA A score		MoCA B score	
		Number	Mean (SD)	Number	Mean (SD)
Age category	18-29	56	23.79 (3.72)	56	26.7 (2.45)
	30-39	52	22.94 (3.54)	53	26.72 (2.6)
	40-49	58	22.12 (3.31)	55	25.27 (3.45)
	50-59	37	20.38 (4.1)	42	23.19 (5.26)
	60-69	53	19.42 (5.02)	55	21.89 (5.37)
	>70	19	16.42 (5.17)	25	19.68 (5.53)
Education category	<12	69	17.48 (4.93)	80	20.21 (5.92)
	12	47	20.91 (2.95)	46	24.5 (3.28)
	>12 and ≤16	91	22.76 (3.67)	88	26.03 (2.61)
	>16	64	24.19 (2.89)	68	26.9 (2.34)

**Table 3:** Multivariate regression table.

	MoCA-A			
	Coef. (SE)	*t*	*P*	95% CI
Age	-0.06 (0.02)	-3.7	<0.0001	-0.09, -0.03
Years of education	0.46(0.04)	10.73	<0.0001	0.37, 0.54
Diabetes	-0.58 (0.58)	-1	0.318	-1.72, 0.56
Hypertension	-0.7 (0.6)	-1.14	0.254	-1.89, 0.5
	MoCA-B			
	Coef. (SE)	*t*	*P*	95% CI
Age	-0.07 (0.02)	-4.48	<0.0001	-0.1, -0.04
Years of education	0.43(0.04)	10.82	<0.0001	0.35, 0.51
Diabetes	-0.07 (0.58)	-0.12	0.91	-1.21, 1.08
Hypertension	-0.7 (0.61)	-1.16	0.25	-1.91, 0.5

## Data Availability

Data is available from corresponding author upon reasonable request.

## Abbreviations

MoCA: Montreal Cognitive Assessment
MoCA-A: Original/standard version of the Arabic MoCA
MoCA-B: Basic version of the Arabic MoCA.
